# Case Report: Short-term response to mesenchymal epithelial transition inhibitor/epidermal growth factor receptor tyrosine kinase inhibitor combination in EGFR-mutated non-small cell lung cancer with acquired MET overexpression after progression and discussion on comprehensive management

**DOI:** 10.3389/fonc.2026.1836074

**Published:** 2026-07-15

**Authors:** Xiaofeng Zhou, Niharika Prasad, Sanjay Jaiswal, Kui Wu, Wenzheng Sun, Li Shen, Lihua Wang, Xueping Xiang, Xiaojuan He, Lin Zheng, Chao-Chao Qin

**Affiliations:** 1 Department of Radiation Oncology, The Second Affiliated Hospital, National Ministry of Education Key Laboratory of Cancer Prevention and Intervention, Zhejiang University School of Medicine, Hangzhou, Zhejiang, China; 2 Department of Conservative Dentistry and Endodontics, Saraswati Dental College, Lucknow, Uttar Pradesh, India; 3 Department of Medicine, Texila American University, Georgetown, Guyana; 4 Department of Radiation Oncology, The Second Affiliated Hospital, Zhejiang University School of Medicine, Hangzhou, Zhejiang, China; 5 Department of Radiology, The Second Affiliated Hospital, Zhejiang University School of Medicine, Hangzhou, Zhejiang, China; 6 Department of Pathology, The Second Affiliated Hospital, Zhejiang University School of Medicine, Hangzhou, Zhejiang, China; 7 Department of Radiation Oncology, Taizhou Cancer Hospital, Taizhou, Zhejiang, China; 8 Oncology Internal Medicine Treatment Unit, Guangyuan Traditional Chinese Medicine Hospital Affiliated to Chengdu University of Traditional Chinese Medicine, Guangyuan, Sichuan, China

**Keywords:** case report, epidermal growth factor receptor tyrosine kinase inhibitor, MET overexpression, non-small cell lung cancer, stereotactic body radiation therapy

## Abstract

Epidermal growth factor receptor tyrosine kinase inhibitor (EGFR-TKI) is a priority and important treatment for EGFR-mutated non-small cell lung cancer (NSCLC) and significantly improves patient’s survival. However, these patients eventually develop disease progression due to acquired resistance to EGFR-TKI. Mesenchymal epithelial transition (MET) gene alteration is one of the common EGFR-independent mechanisms to resist EGFR-TKI. This study reports that a case with chief complaint of dry cough for six months was diagnosed with right lung adenocarcinoma (cT4N3M1c, stage IVB). After first-generation EGFR-TKI treatment, the patient underwent disease progression. After a multi-disciplinary team (MDT) discussion, a re-biopsy and next-generation sequencing (NGS) profiling were performed again, which indicated that the patient harbored the EGFR exon21 L858R mutation and the exon20 T790M mutation. According to the MDT discussion, the patient received the third-generation EGFR-TKI treatment and stereotactic body radiation therapy (SBRT), achieving long-term survival with a high quality of life. After three years of the third-generation EGFR-TKI treatment, the disease progressed again, and a biopsy indicated multiple mutations. After disease progression during the third-generation EGFR-TKI treatment and radiotherapy, the patient received combined treatment with first-generation and the third-generation EGFR-TKI, but could not tolerate the combined treatment. After three courses of chemotherapy plus bevacizumab targeted therapy, the disease continued to progress. Without other effective treatments, the patient received dual-targeted therapy with the third-generation EGFR-TKI and MET-TKI for one month, and the patient experienced a reduction of 51.4% in the size of the lesions in the chest. Unfortunately, the patient developed a lung infection and died of a lung infection. With the assistance of MDT recommendations, the patient received comprehensive treatment and remained asymptomatic, with persistent improvement in the quality of life for 4 years. The overall survival was approximately 56.5 months. Patients with metastatic EGFR-mutated NECLC require multidisciplinary discussions and comprehensive treatment. When the disease progresses, rebiopsy and NGS profiling are required to identify sensitive targeted drugs. Whether MET overexpression is a mechanism for the resistance to third-generation EGFR-TKI in NSCLC patients with EGFR mutations remains to be further investigated.

## 1 Introduction

Non-small cell lung cancer (NSCLC) accounts for approximately 85% of lung cancer cases (1). The IRESSA Pan-Asia Study (IPASS) marked a milestone in the clinical management of NSCLC, pioneering the era of EGFR-targeted therapy (2). EGFR-TKIs significantly improve progression-free survival (PFS) and overall survival (OS) in patients with EGFR-mutated NSCLC (3). However, patients eventually develop disease progression due to acquired resistance.

Mesenchymal epithelial transition (MET) gene is an oncogene located on chromosome 7 long arm (7q21-q31) and encodes a transmembrane receptor tyrosine kinase (4). Hepatocyte growth factor (HGF) binding activates the HGF/MET signaling axis, triggering intracellular cascades that regulate tumor proliferation and metastasis (4). Abnormal activation of the MET is a major bypass resistance mechanism driving EGFR-TKI resistance in NSCLC (5).

Here, we present a case with stage IV NSCLC, who received comprehensive treatment, and achieved a long and high-quality survival. This NSCLC patient with EGFR mutations and MET overexpression achieved a short-term response to a combined treatment of osimertinib and gumarontinib after progression on multiple lines of therapy.

## 2 Case presentation

### Initial treatment

A 62-year-old non-smoking Chinese female peasant without family history of malignancy was admitted to our hospital on July 25, 2019, with a 6-month history of progressive dyspnea and dry cough. Her Eastern Cooperative Oncology Group (ECOG) performance status was 0. This patient had one daughter and two sons. Her families were all in good health. The patient’s right lung had weakened breathing sounds and no rales. The respiratory sounds of the left lung were normal. A swollen right supraclavicular lymph node about 2×2.5 cm in size can be touched, with poor mobility and hard texture. **Figure 1** illustrates the treatment timeline and clinical response.

**Figure 1:**
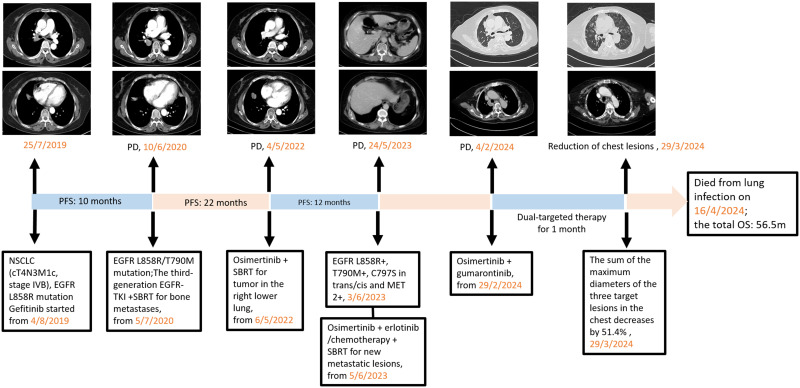
The treatment timeline and clinical response.

- Chest contrast-enhanced computed tomography (CT) on July 25, 2019: a right lower lobe mass adjacent to the oblique fissure, accompanied by bilateral pulmonary, mediastinal and right hilar lymph node metastases (**Figure 2**).
- Single-photon emission computer tomography (SPECT): a metastasis in the left 3rd posterior rib.
- Positron emission tomography-computed tomography (PET-CT): right lower lobe cancer with bilateral lung metastasis, lymph node metastases, right pleural metastasis and bone metastasis (left 3rd posterior rib, right 8th posterior rib) (**Supplementary Figure 1**).
- Bronchoscopy: a protuberant lesion in the right upper bronchus.
- Histopathology and immunohistochemistry (IHC): a poorly differentiated pulmonary adenocarcinoma in the right upper bronchus (**Figures 3A, B**).
- Next-generation sequencing (NGS) (Illumina, Inc., San Diego, CA, USA): EGFR exon21 p.L858R mutation.
- Contrast-enhanced magnetic resonance imaging (MRI): no brain metastasis.
- The patient was diagnosed with right lung adenocarcinoma (cT4N3M1c, stage IVB, the American Joint Committee on Cancer 8^th^ edition), with EGFR exon21 L858R mutation.

**Figure 2:**
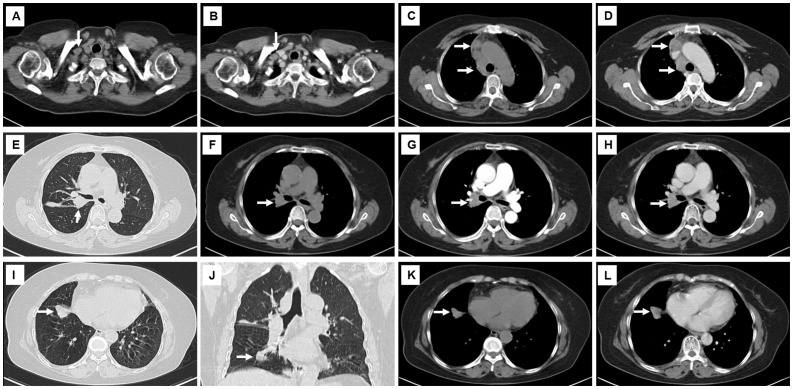
Contrast-enhanced chest CT at initial diagnosis (July 2019). Metastatic lymph nodes in the right clavicular region (white arrow) are shown on chest CT without contrast enhancement **(A)** and contrast-enhanced CT **(B)**, as well as in the mediastinum **(C–D)** and right hilum **(E–H)**. A mass is also present in the right lower lobe adjacent to the oblique fissure and thoracic diaphragm **(I–L)**.

**Figure 3:**
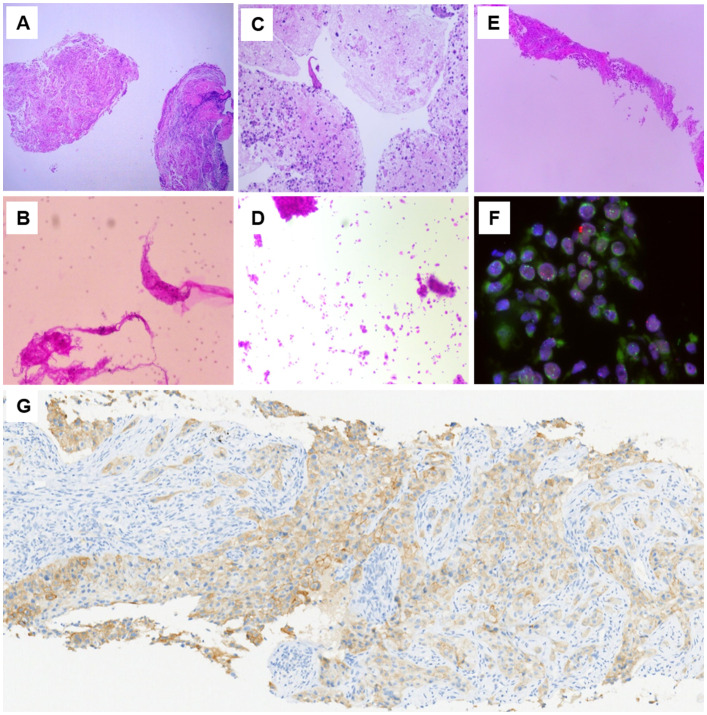
Hematoxylin and eosin staining and FISH results from samples collected at different disease courses. **(A)**, Combined with immunohistochemical (IHC) findings, hematoxylin and eosin staining showed pulmonary poorly differentiated adenocarcinoma in the right upper bronchus in July 2019. **(B)**, EBUS-TBNA of lymph node station 4R in July 2019. **(C)**, Hematoxylin and eosin staining showed pulmonary poorly differentiated adenocarcinoma in the right upper bronchus in June 2020. **(D)**, EBUS-TBNA of lymph node station 10R in June 2020. **(E)**, IHC revealed metastatic lung adenocarcinoma in the right lower chest wall in May 2023. **(F)**, FISH showed no c-MET amplification in June 2023. **(G)**, IHC analysis for c-MET showed c-MET overexpression (2^+^).

We implemented a multi-disciplinary team (MDT) meeting. According to 2019 NCCN guideline of NSCLC and MDT recommendation, the patient received the first-generation EGFR-TKI gefitinib 250mg/day since August 4th, 2019. During the following 10 months, follow-up examination revealed both primary and metastatic lesions had all shrunk and stabilized with only tolerable skin rashes.

### The occurrence of the first disease progression

- In early June 2020, chest CT demonstrated progressive right hilar lymph node metastasis, and SPECT confirmed progression of the left 3rd rib metastasis with a new metastatic lesion in the 4th lumbar vertebra, indicating first disease progression after a 10-month PFS.
- Bronchoscopy, endobronchial ultrasound transbronchial needle aspirations (EBUS-TBNA), histopathology and IHC results of lymph node station 10R: a poorly differentiated metastatic adenocarcinoma (**Figures 3C, D**).
- NGS profiling (NextSeq 500, Illumina, Inc., San Diego, CA, USA): EGFR L858R/T790M mutations, confirming typical EGFR-TKI acquired resistance.

#### Clinical guidelines or supporting evidence

- A multicenter, single-arm phase II clinical study (NCT02981108): almonertinib (HS-10296) had demonstrated favorable efficacy and low toxicity in EGFR T790M-positive NSCLC patients progressing after first-generation EGFR-TKIs, with an objective response rate (ORR) of 66.1% (6).
- The 2020 Chinese Society of Clinical Oncology (CSCO) guideline for NSCLC: almonertinib as a second-line treatment for advanced or metastatic NSCLC with T790M mutation after failure of first- or second-generation EGFR-TKIs (Class II recommendation).
- At that time, osimertinib had not yet been included in the Chinese medical insurance. The case was thoroughly discussed at the MDT meeting again, with the patient and her son also participating.

#### Therapeutic regimen

- The patient agreed to receive almonertinib (110 mg/day) and SBRT from July 5th, 2020.
- SBRT schedule: 20 Gy/2 fractions to the metastasis in the left third posterior rib; 16 Gy/2 fractions to the new metastasis in the fourth lumbar vertebra.
- Four months after taking almonertinib, the patient developed generalized skin rashes, joint swelling and pain, and took a low-dose almonertinib 55 mg/day from September 2020. The patient refused almonertinib due to an intolerable toxicity and took osimertinib 80 mg/day from 24 November 2020. Follow-up examination demonstrated evident shrinking.

### The occurrence of the second disease progression

- The chest contrast- enhanced CT (May 4th, 2022): progression of the tumor in the right lower lobe.
- From July 5th, 2020 (started taking the third-generation EGFR-TKI) to May 4th, 2022, the third-generation EGFR-TKI and SBRT enabled the patients to achieve a 22-month PFS.

#### Clinical guidelines or supporting evidence

- The tumor in the right lower lobe has locally progressed, requiring local treatment according to 2022 NCCN guideline of NSCLC.

#### Therapeutic regimen

- MDT recommendation: the patient continue to take osimertinib;
- SBRT schedule: 50 Gy/5 fractions to this lesion from May 16th, 2022 (**Supplementary Figure 2**). The patient continued to take osimertinib 80 mg/day after SBRT.

### The occurrence of the third disease progression

- Chest contrast-enhanced CT (May 24th, 2023): new metastases in the right lower chest wall and retroperitoneal lymph node.
- Biopsy of the chest wall lesion confirmed metastatic lung adenocarcinoma (**Figure 3E**).
- NGS of 26 genes (DA8600, Shanghai Tongshu Biotechnology Co., Ltd, China): EGFR L858R/T790M/C797S in trans mutations.
- NGS of 25 genes (Illumina NovaSeq 6000, Hangzhou Buping Medical Laboratory Co., Ltd, China) on the same sample: EGFR L858R/T790M/C797S in cis mutations.
- No additional driver gene alterations (MET mutation, amplification, exon 14-skipping, ALK, BRAF, ERBB2, KRAS, RET, etc.) were detected. Insufficient biopsy tissue precluded a third confirmatory test.
- Fluorescence *in situ* hybridization (FISH): MET amplification not detected (**Figure 3F**). MET overexpression was determined by IHC [investigational assay for MET (SP44; Roche Diagnostic Solutions, Tucson, AZ, USA)]. MET overexpression was defined if membranous staining intensity was ≥ 2 in ≥ 50% of tumor cells. IHC result: MET overexpression (IHC 2+) (**Figure 3G**).
- PD-L1 analysis: tumor proportion score of 0%; combined proportion score of 3.

#### Clinical guidelines or supporting evidence

- 2023 NCCN guidelines: no effective standard therapeutic regimen for this patient with EGFR L858R/T790M/C797S mutations.
- 2022 ESMO expert consensus statements on the management of EGFR-mutant NSCLC: the combination of first- and third-generation EGFR-TKIs showed clinical potential for NSCLC patients with T790M/C797S in *trans* mutations (7). This clinical potential was observed in case report (8).

#### Therapeutic regimen

- With MDT discussion, the patient and her families finally chose osimertinib and erlotinib as third-line targeted therapy, plus SBRT for the metastases.
- SBRT schedule: 35 Gy/5 fractions to the metastasis in the right lower chest wall (**Supplementary Figure 3**) and retroperitoneal metastatic lymph node from June 5th, 2023.
- The patient took osimertinib 80 mg/day and erlotinib 150 mg/day to treat the possible clones with T790M/C797S in *trans* mutations. Due to intolerance of a grade 2 rash, the patient stopped taking osimertinib and erlotinib after two months of administration.
- Follow-up contrast-enhanced CT of chest in September 2023 showed disease progression. The patient had an ECOG score of 1.
- The patient was presented to the MDT again. She and families finally chose chemotherapy plus bevacizumab. From October of 2023, the patient received carboplatin and pemetrexed, plus bevacizumab for three cycles. Follow-up examination after three cycles indicated stable disease. The patient could not tolerate and refused chemotherapy. She had a worsening ECOG score of 2.
- PET-CT conducted two months after the chemotherapy (February 7th, 2024): progression of thoracic lesions (**Supplementary Figure 4**) and the presence of multiple brain metastases.
- Whole brain radiation therapy: 30 Gy/10 fractions.
- Due to the patient’s poor mobility and prolonged bed rest, a lung infection occurred. Auscultation revealed moist rales in both lungs.

### Dual-target therapy of osimertinib and gumarontinib

This patient harbored EGFR L858R/T790M/C797S in trans/cis mutations, with MET overexpression (IHC 2+), without definite and effective treatment. A phase III study (9) revealed MET-TKI plus erlotinib improved both PFS and OS in 211 MET-overexpressed (IHC 2+) NSCLC patients. A pooled analysis suggested gumarontinib (a MET-TKI) showed promising efficacy in driver-gene negative locally advanced or metastatic NSCLC patients with MET overexpression (IHC 3+), with an ORR of 37.5% (10). This patient may benefit from the dual-target therapy of third-generation EGFR-TKI plus MET-TKI. With MDT approval and family consent, the patient initiated osimertinib 80 mg daily plus gumarontinib 150 mg daily on February 29, 2024. No adverse toxicities were observed within two weeks, and the gumarontinib dose was escalated to 200 mg daily until April 15, 2024. After one month of dual-targeted therapy, chest CT showed the sum of the maximum diameters of the three target lesions in the chest decreases by 51.4% (**Figure 4**). The reduction of these lesions could not be regarded as a partial response because at that time the patient’s ECOG score was relatively high and a comprehensive examination had not been conducted, which may not rule out the possibility of new lesions appearing. However, the patient still had severely limited mobility and was bedridden, with an ECOG score of 4. The lung infection also became more severe than before. A thorough auscultation of the patient’s lungs reveals diffuse coarse crackles. Imipenem was administered for anti-infection treatment. Ultimately, the patient’s lung infection gradually worsened. The patient’s family gave up the treatment, and the patient died on April 16, 2024. No toxicity related to osimertinib and gumarontinib was observed.

**Figure 4:**
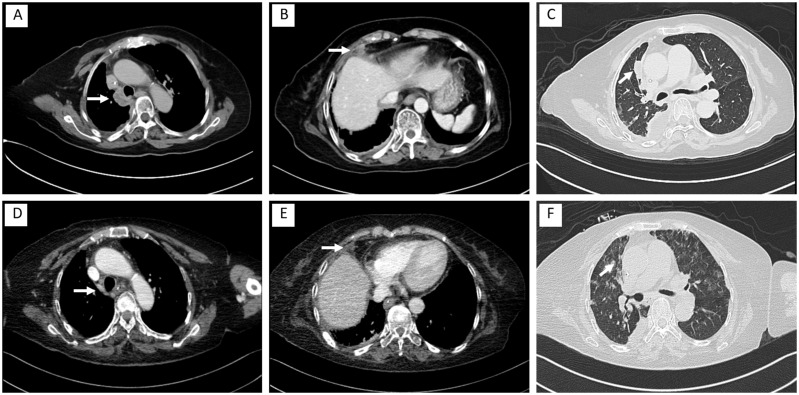
Contrast-enhanced chest CT before **(A–C)** and after **(D–F)** combined targeted therapy with osimertinib and gumarontinib, showing a treatment response. The sum of the maximum diameters of the three target lesions in the chest decreased by 51.4%.

In summary, this patient was diagnosed with NSCLC harboring EGFR L858R mutation on July 30, 2019, and died on April 16, 2024. The patient remained asymptomatic and enjoyed a high-quality life (ECOG 0-1) for 4 years with the assistance of comprehensive treatment. The overall survival was approximately 56.5 months.

## 3 Discussion

We described an NSCLC patient harboring primary EGFR L858R mutation, and secondary EGFRT790M/C797S mutations plus MET overexpression, without MET amplification or exon 14-skipping. For EGFR-mutated NSCLC patients, when resistance to EGFR-TKI occurs, re-biopsy and NGS profiling are critical for identifying resistance mechanisms and guiding individualized treatment regimen for EGFR-TKI-resistant NSCLC. This patient achieved a long survival with high-quality life (ECOG 0-1), mainly due to the following reasons. Firstly, when the patient developed disease progresses, we conducted re-biopsies and NGS, which helped to identify effective treatment regimen. After the third disease progression and biopsy, there was no definite and effective treatment for this patient harboring EGFR L858R/T790M/C797S in trans/cis mutations, with MET overexpression. This patient and her families chose to attempt to receive erlotinib and osimertinib, chemotherapy, as well as osimertinib combined with gumarontinib. Secondly, we successfully implemented a comprehensive treatment approach. When local progression occurred, we recommended targeted therapy combined with SBRT to persistent lesions based on the guidelines, previous studies and MDT discussions. Consistent with previous studies (11, 12), the MDT treatment model significantly improved survival outcomes, with the 56.5-month OS of this patient far exceeding the real-world median OS of 40.60 months for EGFR-TKI-treated NSCLC patients (13).

After 34 months of taking osimertinib, this patient experienced disease progression again and developed EGFR C797S mutation and MET overexpression. The secondary resistance mechanisms to osimertinib can be classified into two categories: EGFR- dependent mechanisms with the C797S mutation being the most common and EGFR-independent mechanisms, such as MET alterations (14–17).

Due to osimertinib binding the C797 residue, the C797S mutation impairs its binding capacity, thus resulting in resistance (18). Recent study has found that 82% of patients develop C797S in *cis* with T790M, while 10% develop it in *trans*, 6% develop C797S alone without T790M, and 2% develop two co-existing C797S clones (one in *cis* with T790M and one in *trans*) (19). C797S/T790M in *cis* or *trans* conformation is important because this conformation can affect the sensitivity of subsequent treatment (20). It was demonstrated that if C797S and T790M occur in *trans*, cancer cells will become resistant to third-generation EGFR-TKIs, but sensitive to a combination of first- and third-generation TKIs. If they are in *cis*, cancer cells will resist any EGFR-TKIs alone or in combination (20). This case was tested in two different laboratories. One laboratory detected an in *trans* mutation, while the other detected an in *cis* mutation.

The possible reasons for these two different test results are as follows. Firstly, different subclonals and biopsy samples, regarded as tumor heterogeneity, may lead to different test results. Zhao et al. reported that resistance to osimertinib is highly heterogenous not only among different individuals but also within a single patient (21). This patient received erlotinib plus osimertinib after developing resistance to osimertinib. However, she developed disease progression two months after taking erlotinib plus osimertinib. This might suggest the proportion of the clones with C797S in *trans* mutation in our case was relatively low. Short-term administration of erlotinib plus osimertinib for two months may resulted in the therapeutic effect might not have yet manifested. It seems that the clones with C797S in *trans* and in *cis* mutation can coexist in a single patient. Secondly, different detection platform, technique and reagent in our report may lead to different test results from two laboratories. The detection platforms, techniques and reagents used in the two laboratories had been certified. It’s difficult to determine which laboratory’s test result was accurate in a single patient. Actually, we couldn’t re-test the C797S mutation in the third laboratory due to the lack of sufficient samples. Among several possible influencing factors, we are inclined to believe tumor heterogeneity may play an important role and resistant mechanisms of C797S in *trans* and *cis* against osimertinib coexist. However, it’s necessary to interpret the results of C797S mutation in this case cautiously.

MET overexpression can arise from MET amplification, mutation or transcriptional enhancement (22). MET fusion, point mutation, amplification and exon 14-skipping were not detected in both tests after the third biopsy. FISH, considered the gold standard for the detection of MET amplification (23), did not detect MET amplification (**Figure 2E**). Therefore, MET overexpression was independent of MET amplification or exon 14-skipping in the present case.

The MET overexpression have recently emerged as a potential target for targeted therapy. The combination of EGFR-TKI and MET inhibitor improved the OS and PFS of patients with MET overexpression (IHC ≥ 2+) (9, 24). Gumarontinib, a highly selective MET-TKI, demonstrated a favorable safety profile and promising efficacy (10, 25, 26). The present case chose to attempt the dual-targeted therapy of osimertinib and gumarontinib for one and half a month. Although chest CT showed a reduction of 51.4% in the size of the lesions in the chest after one month of dual-targeted therapy, it’s difficult to determine whether the dual-targeted therapy caused the lesions to shrink. For EGFR-mutant NSCLC patients with MET overexpression, dual-targeted therapy of EGFR-TKI with MET inhibitors may be worthy of further investigation and has potential feasibility. This case report may provide possible hints for the treatment of EGFR-mutated NSCLC after resistance to third-generation EGFR-TKI. It’s necessary to explore the resisting third-generation EGFR-TKI mechanism related to MET overexpression, as well as how the MET gene signaling pathway interacts with the EGFR signaling pathway to develop this resistance.

We recognize several limitations in this study. Firstly, it was a case report with potential for bias. This limitation reminds us that the results should be interpreted with caution. Secondly, since the dual-targeted therapy of osimertinib and gumarontinib was only administered for one month and a half, it was not sufficient to assess the efficacy and toxicity of it. The therapeutic effect and toxicity need to be evaluated over a longer period of time. Thirdly, the interval between the dual-targeted therapy of osimertinib plus gumarontinib and the previous treatment was too short to assess the efficacy of this dual-targeted therapy. Finally, as a case report, we didn’t validate the function of MET overexpression. To reduce these biases and limitations, it is necessary to conduct further investigation.

## 4 Conclusion

For patients with stage IV EGFR-mutated NSCLC, the first-line treatment is undoubtedly EGFR-TKI targeted therapy. When they developed disease progresses, we need to conduct re-biopsies and NGS, which helped to identify effective targeted therapy regimen. EGFR exon20 T790M/C797S mutations are the common secondary resistance mechanisms. Resisting third-generation EGFR-TKI mechanism related to MET overexpression remains to be further investigated. Due to the limitations, we should interpret these preliminary results cautiously. Additional research is warranted to evaluate the safety and efficacy of dual-targeted therapy with osimertinib and gumarontinib for EGFR-mutated NSCLC patients harboring secondary MET overexpression.

## Data Availability

The datasets presented in this study can be found in online repositories. The names of the repository/repositories and accession number(s) can be found in the article/**Supplementary Material**.
