# Supplementary figures and images for: Case Report: Short-term response to mesenchymal epithelial transition inhibitor/epidermal growth factor receptor tyrosine kinase inhibitor combination in EGFR-mutated non-small cell lung cancer with acquired MET overexpression after progression and discussion on comprehensive management
Source: Front Oncol. 2026 Jul 15;16:1836074. doi: 10.3389/fonc.2026.1836074 (PMC13416089; doi:10.3389/fonc.2026.1836074)

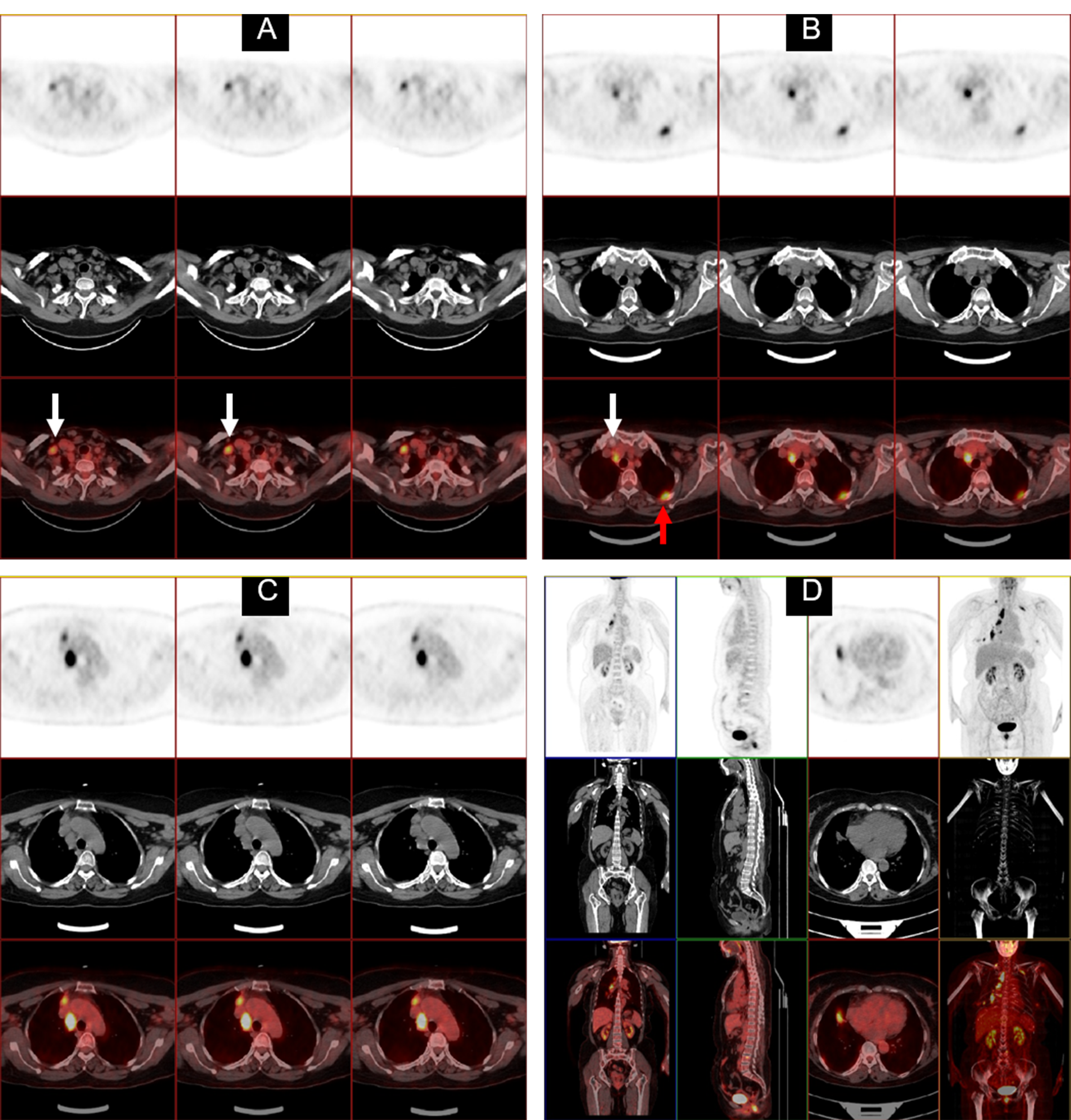

Supplement: Supplementary file 1 [file Image1.tif]

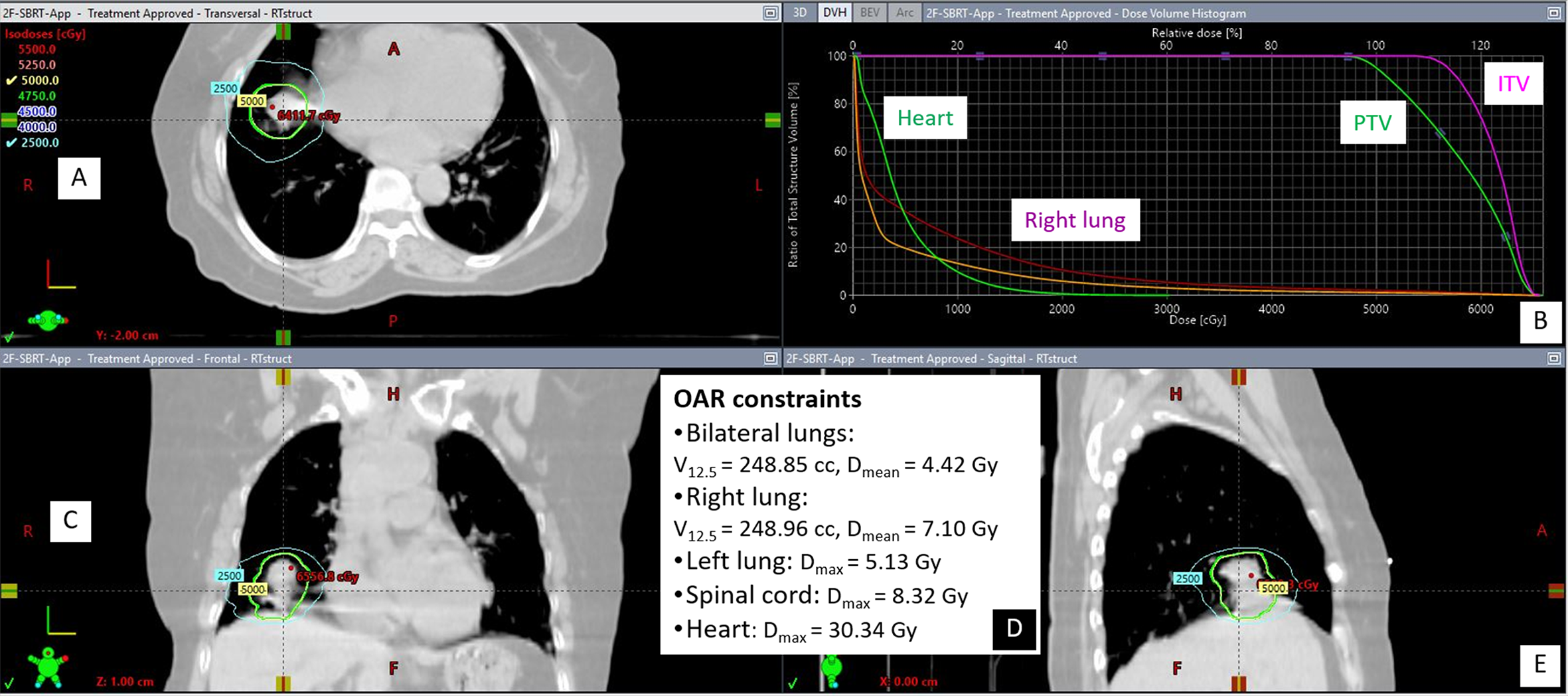

Supplement: Supplementary file 2 [file Image2.tif]

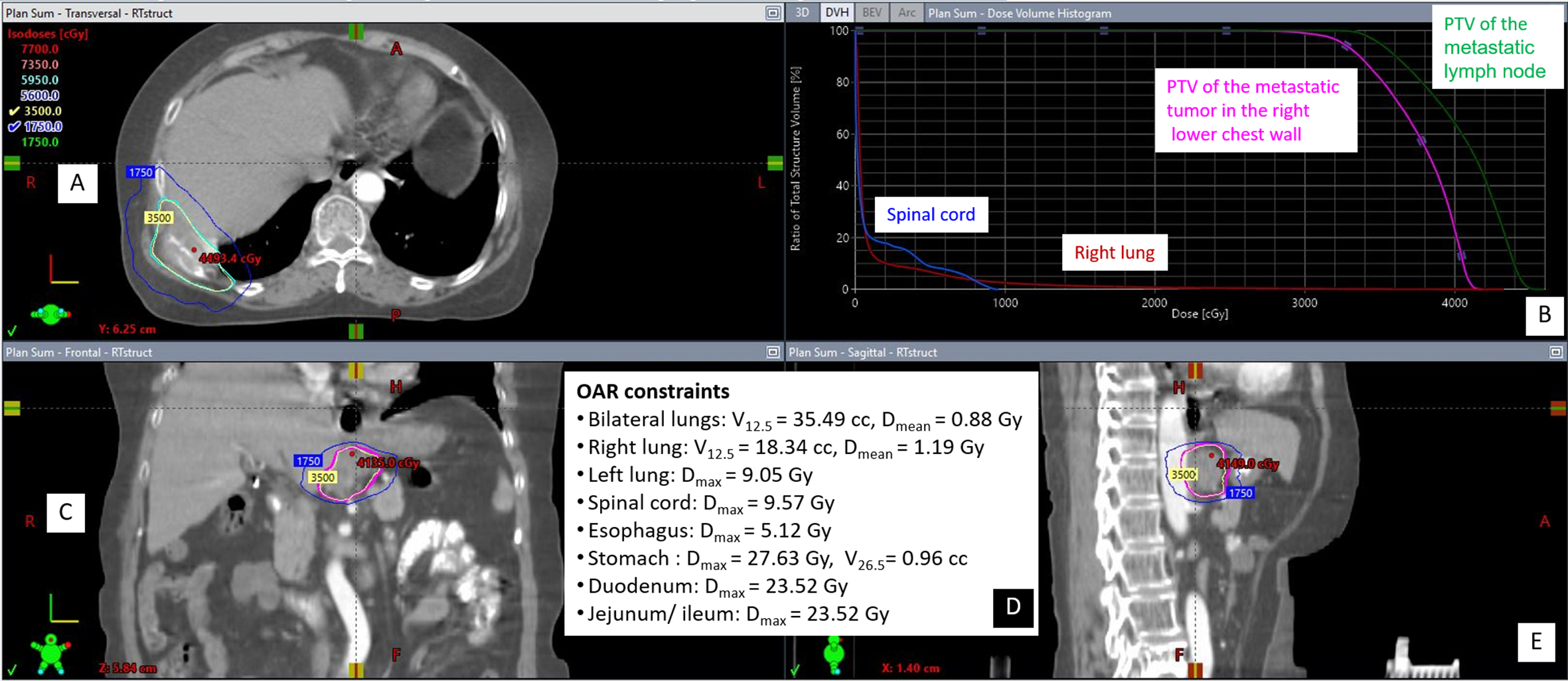

Supplement: Supplementary file 3 [file Image3.tif]

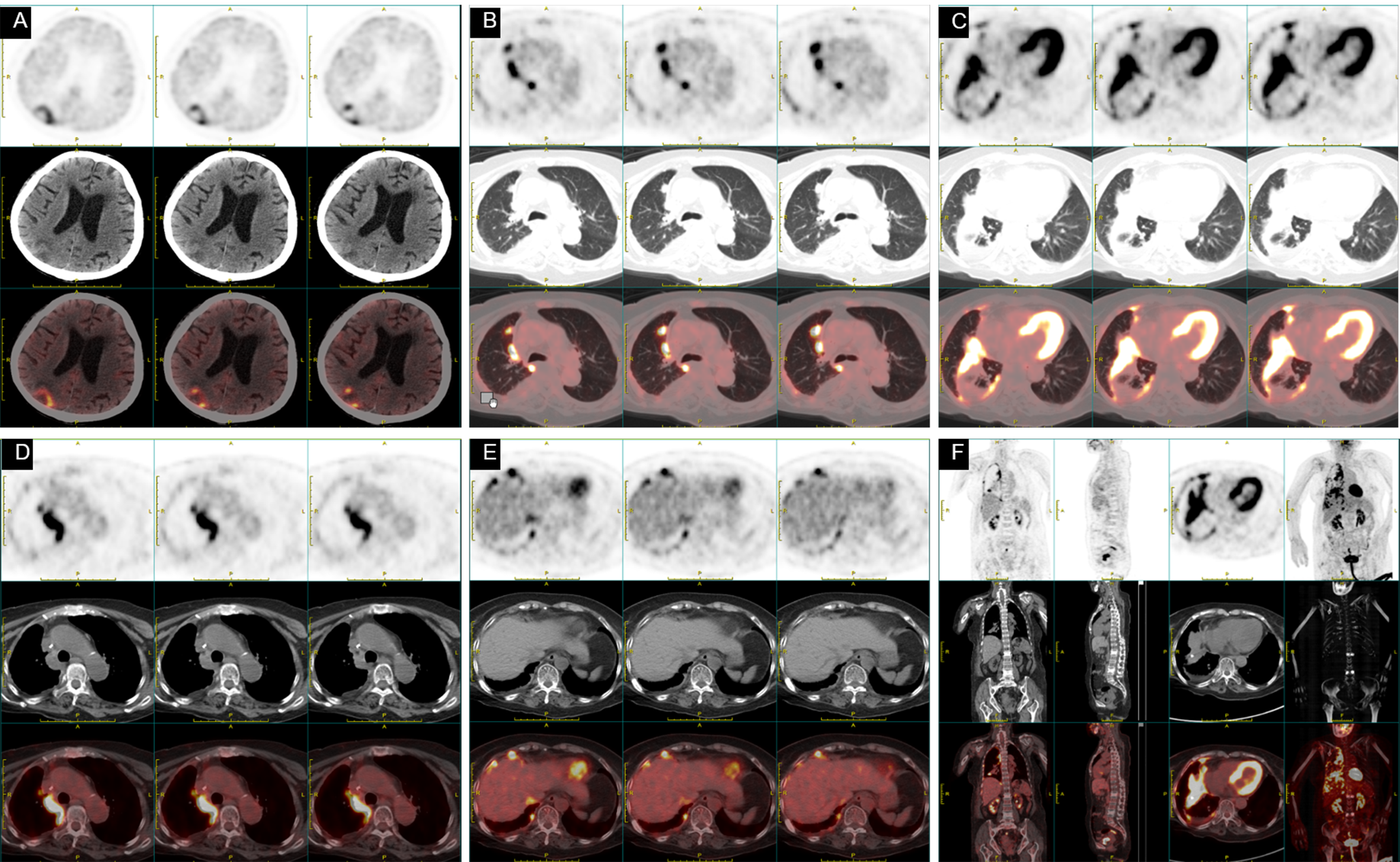

Supplement: Supplementary file 4 [file Image4.tif]
